# An Infected Urachal Cyst With Umbilical Granuloma in an Adult Patient

**DOI:** 10.7759/cureus.70559

**Published:** 2024-09-30

**Authors:** Naomi M Watkins-Granville, Saumil Parikh, Tyler Liguori, Ravikumar Brahmbhatt, Antonios Tsompanidis

**Affiliations:** 1 General Surgery, CarePoint Health-Christ Hospital, Jersey City, USA; 2 General Surgery, Rowan-Virtua School of Osteopathic Medicine, Stratford, USA; 3 Graduate Medical Education, CarePoint Health-Christ Hospital, Jersey City, USA; 4 Graduate Medical Education, CarePoint Health-Bayonne Medical Center, Bayonne, USA

**Keywords:** adult urachal cyst, infected urachal cyst, intravesical urachal cyst, umbilical granuloma, urachal anomalies

## Abstract

The urachus, an embryonic organ associated with the bladder, typically undergoes degeneration shortly after birth. Inadequate closure of the urachus can result in urachal malformations, with the most common being a urachal cyst. Failure to promptly identify and address this condition can lead to complications such as sepsis, fistula development, and cyst rupture, mimicking symptoms of peritonitis. Therefore, maintaining a heightened level of suspicion is crucial for timely diagnosis and effective management of urachal cysts, particularly in emergency room settings. We present the case of a 24-year-old male who exhibited clinical signs consistent with an acute abdomen. The use of imaging techniques confirmed the presence of an infected urachal cyst, which was successfully treated through surgical intervention.

## Introduction

Urachal cysts, part of a spectrum of urachal abnormalities typically encountered in infancy, are notably rare in adults [1]. An embryologic remnant formed through the obliteration of the allantois, the urachus normally degenerates after birth, leaving behind the median umbilical ligament [2,3]. Incomplete degeneration of the urachus can result in urachal cysts, a congenital anomaly seen infrequently in children and even more rarely in adults.

The specific etiology and occurrence of urachal cysts in adults remain largely unknown, and patients may remain asymptomatic for years. Urachal cysts are often detected only when they become infected, causing the patient to develop constitutional symptoms and umbilical discharge [4,5]. Typical symptoms include umbilical discharge, abdominal pain, and signs that mimic cystitis, such as dysuria [5,6].

We present a compelling case of the acute presentation of a urachal cyst in an adult male, which was successfully diagnosed and managed. The rarity of urachal abnormalities in adults, compounded by nonspecific symptoms, underscores the significance of this case. We describe the presentation of a urachal cyst in an otherwise healthy adult male, characterized by a tender abdomen and purulent umbilical discharge, a clinical scenario requiring thorough investigation. Additionally, the resolution of this case using robotic surgery highlights the effectiveness of this minimally invasive approach. This case report adds to the limited body of literature on this intriguing clinical entity.

## Case presentation

A 24-year-old male with no significant medical, surgical, or social history presented to the emergency department with a five-day history of periumbilical pain, erythema, swelling, and “discharge from the belly button.” Associated symptoms included fever and pain with urination. The patient reported no injury or trauma to the abdomen. He also denied nausea, vomiting, penile pain, penile discharge, and scrotal pain or swelling. The patient had been taking tylenol for his pain, which provided minimal relief.

**Figure 1:**
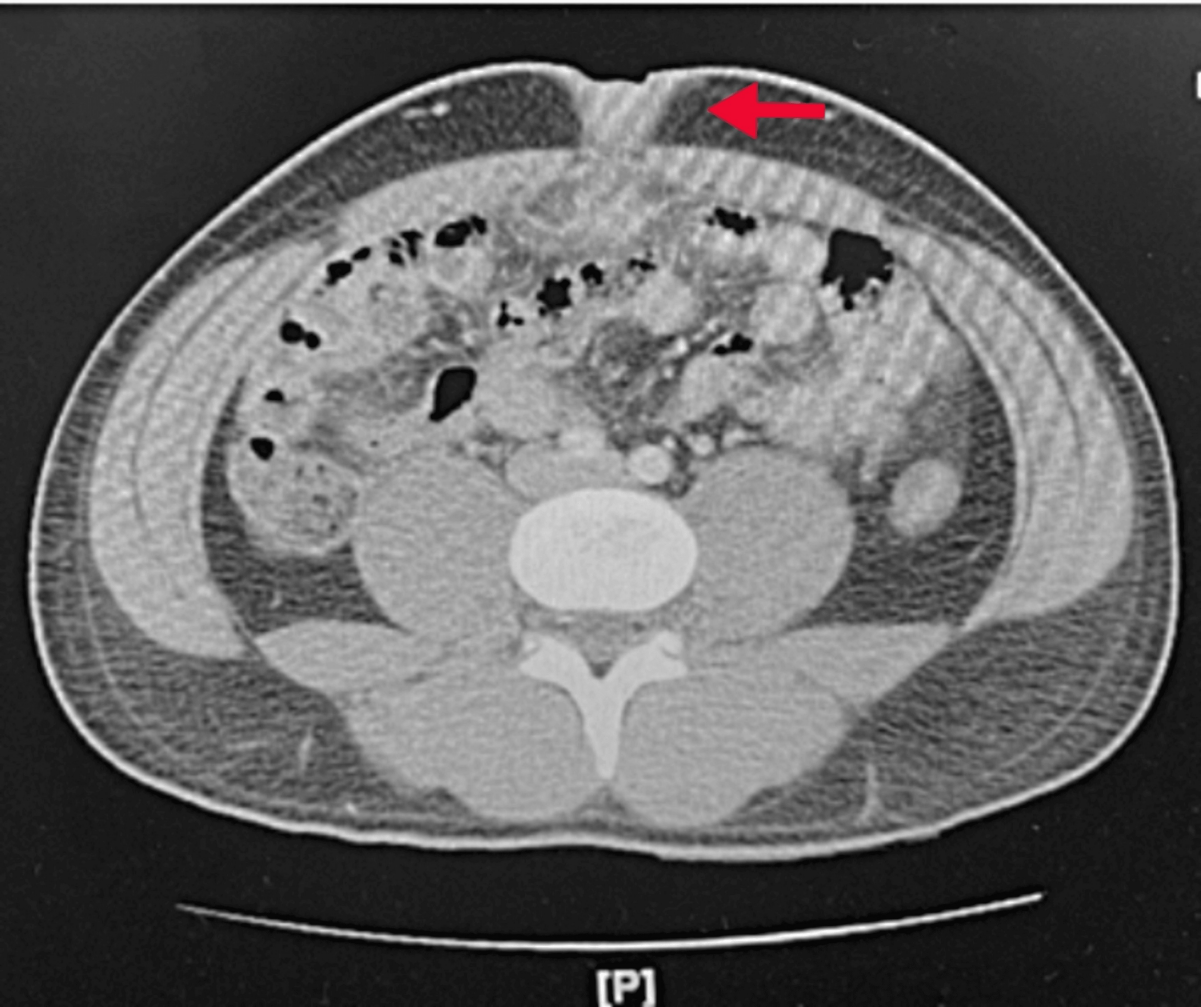
Transverse section CT abdomen pelvis

**Figure 2:**
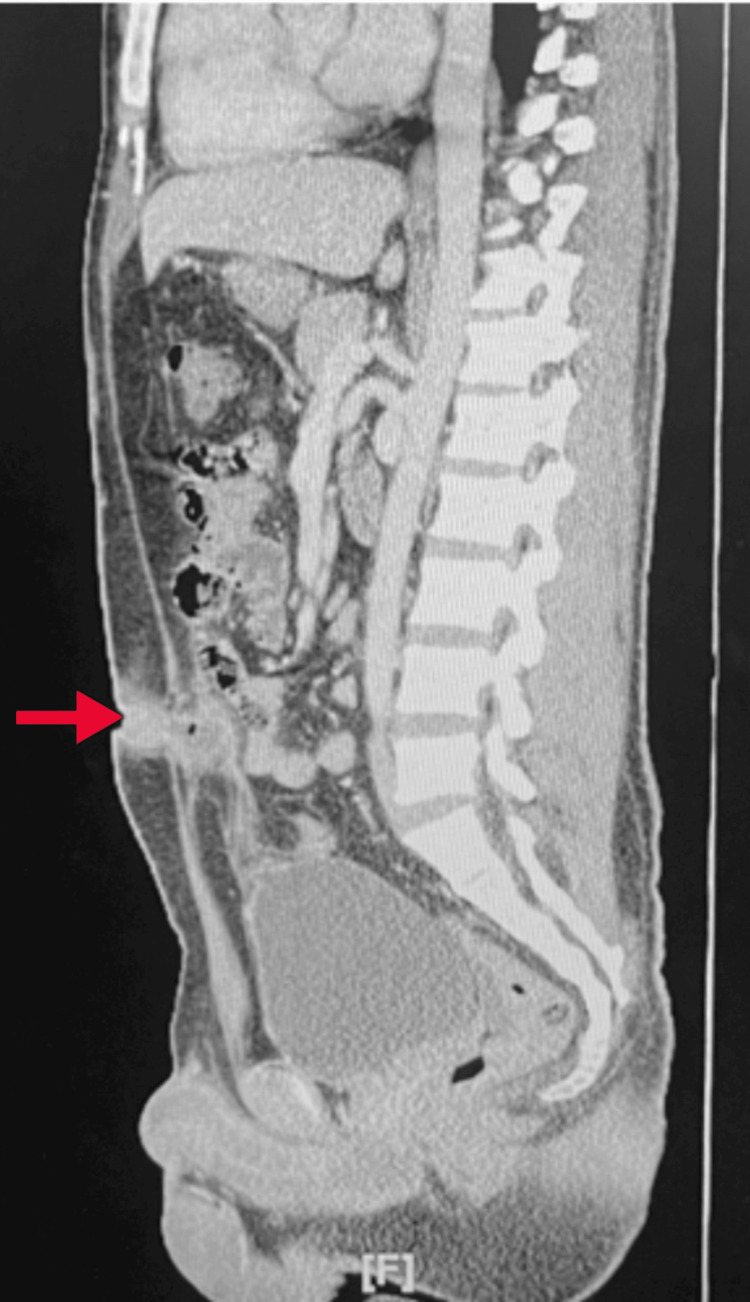
Sagittal section CT abdomen pelvis

A review of systems was consistent with the initial presentation of fever, chills, pain with urination, and pain in the umbilical area. Initial vital signs were unremarkable, aside from a temperature of 37.3°C/99.2°F. Abdominal examination revealed periumbilical pain and purulent drainage from the umbilical fossa, consistent with the initial presentation. Laboratory results, including a complete blood count (CBC), basic metabolic panel (BMP), and urinalysis (UA), were unremarkable. Computerized tomography (CT) of the abdomen and pelvis showed a 1.8 cm x 2 cm abscess in the anterior abdominal wall, posterior and inferior to the umbilicus or rectus sheath region. Some inflammation was noted around the area, but no abnormalities were observed in the bladder (Figure 1 and Figure 2). Culture of the wound discharge confirmed the presence of Corynebacterium species.

The patient was admitted to the hospital and started on empiric antibiotics, piperacillin/tazobactam and vancomycin. The patient's pain was managed with tramadol, acetaminophen, and ketorolac as needed. Urology and general surgery were consulted.

A cystogram, performed at the recommendation of urology, revealed a urachal remnant and/or urachal cyst. Slight residual contrast in the urachal remnant remained post-drainage. No abnormalities were noted in the bladder. The patient was subsequently scheduled for surgery, which was performed four days after the initial presentation to the emergency room. The operative course proceeded as follows.

Pre-operative diagnosis

Umbilical granuloma and urachal cyst were suspected before the surgery.

Operative findings

The findings included an umbilical granuloma with copious pus drainage, suspicious for infection; urachal cysts; umbilical hernia; omental adhesions; and a patent bladder with no extravasation.

Surgery performed

A 3 cm x 2 cm umbilical granuloma was excised, and a 2 cm x 1 cm umbilical hernia was identified. A large urachal cyst surrounding the umbilical area between the bladder was demarcated and excised using a robotic-assisted laparoscopic technique. Bladder irrigation with methylene blue was performed following the excision of the urachal cyst, which showed no leakage. An umbilical hernia repair with primary closure was also performed laparoscopically with robotic assistance. A 19FR BLAKE drain was placed and secured to the skin. All ports from the robotic-assisted laparoscopic procedure were removed, and the umbilical wound site was packed. The port sites were closed appropriately before applying a sterile dressing. The umbilical granuloma and urachal cyst were removed as specimens and sent to pathology.

The patient tolerated the surgery well and experienced no apparent complications. The umbilical packing was changed on post-op days one through three. The patient was discharged on post-op day three with oral amoxicillin/clavulanate (augmentin 875 mg-125 mg), to be taken twice daily for seven days. At the time of discharge, the packing gauze was removed from the umbilical wound, and the area was covered. The patient was advised to follow up with the surgical team in the outpatient setting for post-op evaluation and drain removal. The patient's drain was successfully removed during a follow-up visit, and there have been no reports of complications or recurrence.

Pathology report

The pathology sample showed fibrotic tissue with squamous and focal urothelial lining consistent with a urachal cyst. Acute and chronic inflammation and necrosis were also noted.

## Discussion

The approximate incidence of urachal anomalies is 1 in 5000 [4]. Urachal cysts are the second most common urachal anomaly. Urachal cysts can be asymptomatic; therefore, they may also be incidental findings following abdominal imaging or during abdominal surgery. The incidence of urachal cysts is possibly under-reported due to a lack of symptomatology. Similar to most adults who present with urachal cysts [5,7], this patient was asymptomatic prior to developing discharge from the umbilicus, which raised high suspicion for infection. The most common bacteria that infect a urachal cyst are *Staphylococcus aureus*, although other organisms such as *Escherichia coli*, *Enterococcus faecium*, and *Klebsiella pneumoniae *have been documented [8,9]. In this patient, the culture was positive for *Corynebacterium* species. The variety of organisms found suggests that culturing the fluid from the umbilicus is an important step in diagnosing and treating urachal cysts. This ensures administration of the appropriate antibiotic and reduces the risk of sepsis, a potential complication of untreated urachal cysts [9,10].

While symptomatic urachal anomalies occur more frequently in children, they can also present in adults with symptoms that mimic those of cystitis, obstructed hernia, or other disorders causing acute abdominal pain [11]. Therefore, imaging of the abdomen and pelvis is important to distinguish a urachal cyst from other abnormalities presenting similar symptoms. The tendency for urachal cysts to present with nonspecific symptoms most commonly results in their discovery via initial CT imaging in the process of ruling out other diagnoses [12]. In this case, further imaging was ordered after CT revealed an abscess. It is important to note, however, that ultrasound is the preferred method for diagnosing urachal cysts due to its ability to visualize extra-peritoneal structures. Additionally, a connection to the bladder can be determined without additional urological testing, such as cystoscopy (a customary practice), or exposure to unnecessary radiation [12]. While using more sensitive and conservative imaging would require a high level of suspicion for a urachal cyst, it would allow for more prompt treatment.

Two methods for urachal cyst treatment have been reported in the literature. The first method is a “two-stage approach,” which involves antibiotics and the potential resolution of the infection prior to surgical management. The second method entails excision of the cyst without waiting for infection resolution. While the evidence is limited, it has been suggested that post-operative complications are decreased with the “two-stage approach” [5,13]. Treating our patient with the “two-stage approach” resulted in no complications, and the patient tolerated the treatment well. However, it must be noted that delaying excision in more serious infections could result in rupture of the cyst, peritonitis, sepsis, or fistula formation [10]. Therefore, closely monitoring vital signs, laboratory results, and patient presentation is imperative once the diagnosis of a urachal cyst is made.

Historically, urachal cysts have been managed conservatively, through open surgery, or laparoscopic (both non-robotic- and robotic-assisted) surgery. Conservative management with only aspiration and antibiotic therapy results in about a 30% recurrence rate for symptomatic urachal cysts, such as the one presented in this case [14]. Surgical removal of the urachal cyst is considered the most definitive treatment. Open urachal cyst removal is performed through a lower midline laparotomy or infraumbilical incision, which can involve the removal of the umbilicus [15]. A laparoscopic approach is becoming more widely accepted for urachal cyst removal due to its minimally invasive nature. Compared to open surgery for a urachal cyst, post-operative pain is reduced, morbidity is lower, and a quicker recovery time is noted [15,16]. Furthermore, our patient is younger and has no prior abdominal surgical history, making better cosmesis in laparoscopic surgery over open surgery particularly desirable [17].

In terms of robotic-assisted laparoscopic urachal cyst excision versus non-robotic surgery, the decision was made based on the aforementioned points, with the added benefit of better visualization of the planes of dissection and the use of more dexterous instruments.

## Conclusions

The diverse clinical presentation of urachal anomalies in adults poses a diagnostic challenge that may lead to misdiagnosis. The importance of early detection cannot be overstated, as timely diagnosis plays a crucial role in planning and implementing appropriate medical and surgical interventions. This case report highlights the uncommon nature of this congenital anomaly and emphasizes the need for health care professionals to be aware of the condition. By contributing to the existing literature, we aim to improve the ability to promptly identify and treat urachal anomalies using minimally invasive surgical techniques, ultimately reducing the morbidity associated with delayed recognition and treatment.
